# *Pancreas*++: Automated Quantification of Pancreatic Islet Cells in Microscopy Images

**DOI:** 10.3389/fphys.2012.00482

**Published:** 2013-01-03

**Authors:** Hongyu Chen, Bronwen Martin, Huan Cai, Jennifer L. Fiori, Josephine M. Egan, Sana Siddiqui, Stuart Maudsley

**Affiliations:** ^1^Receptor Pharmacology Unit, Laboratory of Neuroscience, National Institute on Aging, National Institutes of HealthBaltimore, MD, USA; ^2^Metabolism Unit, Laboratory of Clinical Investigation, National Institute on Aging, National Institutes of HealthBaltimore, MD, USA; ^3^Diabetes Section, Laboratory of Clinical Investigation, National Institute on Aging, National Institutes of HealthBaltimore, MD, USA

**Keywords:** pancreas, islets of Langerhans, alpha-cells, beta-cells, quantification, software, algorithm

## Abstract

The microscopic image analysis of pancreatic Islet of Langerhans morphology is crucial for the investigation of diabetes and metabolic diseases. Besides the general size of the islet, the percentage and relative position of glucagon-containing alpha-, and insulin-containing beta-cells is also important for pathophysiological analyses, especially in rodents. Hence, the ability to identify, quantify and spatially locate peripheral, and “involuted” alpha-cells in the islet core is an important analytical goal. There is a dearth of software available for the automated and sophisticated positional quantification of multiple cell types in the islet core. Manual analytical methods for these analyses, while relatively accurate, can suffer from a slow throughput rate as well as user-based biases. Here we describe a newly developed pancreatic islet analytical software program, *Pancreas*++, which facilitates the fully automated, non-biased, and highly reproducible investigation of islet area and alpha- and beta-cell quantity as well as position within the islet for either single or large batches of fluorescent images. We demonstrate the utility and accuracy of *Pancreas*++ by comparing its performance to other pancreatic islet size and cell type (alpha, beta) quantification methods. Our *Pancreas*++ analysis was significantly faster than other methods, while still retaining low error rates and a high degree of result correlation with the manually generated reference standard.

## Introduction

Recent research has demonstrated that the maintenance of coherent somatic metabolism is vital for protecting against age or disease-related central and peripheral pathophysiology (Martin et al., 2008, 2010, 2012; Cai et al., 2012; Siddiqui et al., 2012). A large proportion of somatic metabolism is controlled by the regulated uptake and metabolism of the primary caloric foodstuff, i.e., glucose. Therefore, an appreciation of how somatic energy function is altered in aging or pathophysiological states entails at some point an in-depth analysis of the insulinotropic glucose-regulatory system. This system is centered upon the pancreas, a large secretory organ possessing endocrine secretory cells that release insulin into the major circulation in response to dietary glucose. The insulin-releasing cells, termed beta-cells, are situated into sub-organ cellular clusters termed Islets of Langerhans. The growth, development, function, and sensitivity of these beta-cells is, in-part, managed via a local secretory interaction with glucagon-containing alpha-cells that are also present in the pancreatic islets (Jain and Lammert, 2009). Islets also contain several other secretory cell types that are responsible for the local and systemic release of somatostatin (delta cells), pancreatic polypeptide (PP cells), and ghrelin (epsilon cells). However the majority of the pancreatic islet mass is made up of beta- (65–80%) and alpha-(15–20%) cells and thus, these cell populations are the most consistently measured to assess the connection between islet morphology and pancreatic function.

Rodent models are currently the most widely used experimental animal models. The pancreatic islets of rodents possess a distinct pattern in the relative islet distribution of alpha- and beta-cells. Hence, in rodents the central core of the islet comprises a near pure mass of beta-cells while in normal functioning islets the smaller numbers of alpha-cells are excluded from the beta-cell core and are found in a peripheral formation encircling the islet. Multiple studies have demonstrated that there are considerable correlations between the intra-islet physical distribution and interaction of these two cell types (alpha and beta) and somatic energy metabolic function (Van Assche et al., 1978; Parsons et al., 1992; Sorenson and Brelje, 1997, 2009; Karnik et al., 2007; Huang et al., 2009). One of the most common findings in the pancreatic islets, in states of metabolic dysfunction, is the abnormal presence of alpha-cells within the beta-cell islet core. The aberrant presence of these cells is often referred to as alpha-cell involution. As such, the visual analysis of these two important cell types within immunostained endocrine pancreatic islets, may help scientists develop a deeper understanding of etiology of metabolic diseases such as obesity and diabetes mellitus (Gepts, 1965; Clark et al., 1988; Sreenan et al., 1999; Sherry et al., 2006; Marchetti et al., 2008; Matveyenko and Butler, 2008) and how this is associated with morphological cellular pancreatic signatures. In particular, quantification of involuting alpha-cells (in addition to total changes in alpha- or beta-cell mass) that invade the interior of an islet is important for the detection of pancreatic abnormalities. Unfortunately, even with expert immunohistochemical staining, microscopic imaging of sectioned pancreata, with insulin (beta-cell) and glucagon (alpha-cell) detection can still generate visually noisy images that are difficult to interpret consistently and impartially. The varied use of different microscopic instruments and immunohistochemical staining procedures can compound these visual inconsistencies. Due to the inevitable inclusion of pixel noise, a naïve quantification of red and green pixels is potentially insufficient for accurate pancreatic islet structural analysis. To address these issues, we have developed a novel software application for the complete sub-islet automation of alpha- and beta-cell positional quantification and analysis, from general fluorescent microscopic images. Additionally, our novel software program can also analyze large batches of pancreatic immunohistochemistry images and provide accurate quantitative data on islet area, percentage of beta-cells, percentage of alpha-cells, and percentage of involuted alpha-cells. Our algorithm requires no manual intervention, and is resilient against noise, and therefore produces high-accuracy, high-speed image analysis. To assess our accuracy and high-speed processing we performed extensive computational validations. We assessed how our program demonstrates so-called “image resilience” by contending with image noise, i.e., the natural grainy texture of images. Images can often possess trace amounts of whitespace within a pancreatic islet and trace amounts of staining away from the islet. Resiliency to this type of image noise was validated by manually examining the outline of a cell and comparing it to the outline delineated by the active contour model. The model closely, if not exactly, matched nearly every image in the testing set used for validation. Therefore we feel that *Pancreas*++ represents an important and robust addition to current techniques for pancreatic image analysis and will hopefully assist in the investigation of connections between islet morphology and disease.

## Materials and Methods

### Islet detection using active contour models

The first step in our methodology for automated analysis involves islet detection for the localized quantification of alpha- and beta-cells within islets. Focusing analysis on each individual islet allows for the discarding of free-floating red and green pixels independent of any pancreatic islet. Using a combination of thresholding, nearest-neighbor interpolation, and active contour models, large contiguous regions, i.e., the islets of interest, can be easily extracted without user interaction (Kass et al., 1988; Cohen, 1991). A crucial advantage of this method is the ability to fill in large amounts of space within pancreatic cells, a common product of noise in microscopic image analysis. Whereas conventional histogram analysis would fail to address this problem, islet detection using active contour models allows for interior spaces within an islet to influence total islet area calculation.

A popular model of choice for delineating outlines from a noisy image is the active contour model (Cootes et al., 1995). Active contours are represented by a dynamic “spline,” or collection of points (*v*), that bends and iteratively evolves under the influence of internal and external forces. The contour attempts to find the orientation that minimizes the energy function originating from the snake itself (interior), and from image forces (exterior). The function being minimized is as follows:

$$\begin{array}{l} \int_{0}^{1}E_{\text{internal}}\left(v\left(s\right)\right)+E_{\text{image}}\left(v\left(s\right)\right)ds\\ v=\left\{\left(x_{0},y_{0}\right),\left(x_{1},y_{1}\right),\left(x_{2},y_{2}\right),\ldots ,\left(x_{n},y_{n}\right)\right\} \end{array}$$

*E*_internal_ is composed of the weighted sum of two elements: *E*_continuity_ and *E*_curvature_. These two forces place limitations on the snake’s ability to stretch and bend, respectively. For the purposes of our algorithm, these two functions are defined as:

$$\begin{array}{l} E_{\text{continuity}}=\alpha \left(s\right){\left\|\frac{dv\left(s\right)}{ds}\right\|}^{2}\\ E_{\text{curvature}}=\beta \left(s\right){\left\|\frac{dv^{2}\left(s\right)}{ds^{2}}\right\|}^{2} \end{array}$$

*E*_continuity_ attempts to minimize the distance between the contour’s points, having the added effect of causing the contour to shrink. To encourage smoothness and avoid oscillations, *E*_curvature_ penalizes high curvatures. Both continuity and curvature are approximated by finite differences applied to the contour’s points. Finally, *E*_image_ is the force that pushes the spline to various features of the image. Since we are interested in delineating the surface of the pancreatic islets, *E*_image_ must reach a minimum at the image’s edges. After applying a gradient transform on the image, *E*_image_ is simply the negative value of the intensities of every pixel:

$$E_{\text{image}}=-{\left|\nabla I\right|}^{2}$$

An important issue regarding the active contour is the initialization of contour points. This is achieved by standard thresholding and nearest-neighbor interpolation to remove noise outside of the islets and fill tissue gaps within islets. A flood fill algorithm is used to determine contiguous regions within the image, and, for each region sufficiently large, we initialize an active contour as a collection of points delineating a rectangle encompassing the entire islet. The contour is then allowed to deform into the shape of the contained islet.

We performed parameter optimization for our *Pancreas*++ algorithm as follows. Briefly, for algorithm parameter optimization we employed a subset of the overall testing image set. With this specific subset we honed the functional parameters of *Pancreas*++ with the use of manual quantification of alpha-cells (by count) as the “*gold standard*” of quantification. We used a grid search on the following parameters using the ranges specified while maximizing the accuracy with respect to the “*gold standard*” quantification: step size (3–9); continuity coefficient (0.0–3.0); connectivity coefficient (0.0–3.0); image coefficient (0.0–3.0); nearest neighbors for interpolation (0–100); intensity threshold (0–255).

### Alpha- and beta-cell quantification

**Figure 1:**
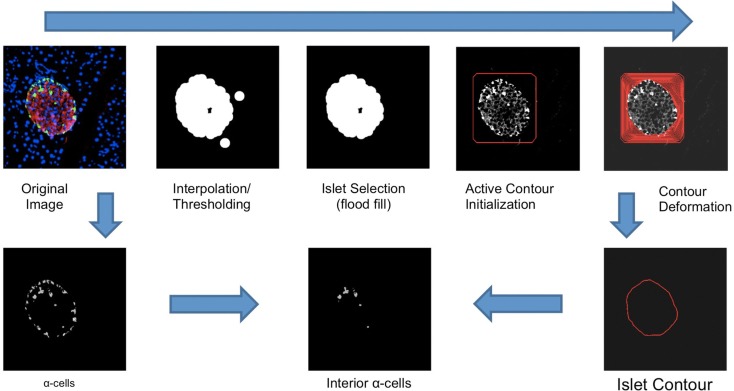
**An illustration of the workflow of the *Pancreas*++ algorithm for determining interior alpha-cells**. Contour deformation was run at 800 iterations. The islet contour was generated with linear interpolation of contour points. Interior alpha-cells were determined by the distance between each alpha-cell centroid and the nearest point in the surrounding islet contour.

Alpha-cell regions can be isolated by the use of color thresholding to generate binary masks. Each individual cell can be isolated by counting the contiguous regions via a flood fill algorithm. Since we are only interested in alpha- and beta-cells within islets instead of background noise, we only consider red and green pixels within the interior of the active contours outlined above. First, linear interpolation is applied to the collection of points in each active contour to produce a polygon outlining the shape of each islet. A ray-casting algorithm is used to determine, for each alpha-cell, whether or not its centroid lies within the interior of an islet. All cells exterior to every islet are assumed to be noise and discarded. At this point, quantification of total alpha-cells can be computed by summing the areas of each contiguous region in the alpha-cell binary mask within each islet. Since each islet’s area can be computed with the active contours delineating each islet, beta-cell area is trivially computed by the difference between total islet area and alpha-cell area. In order to disambiguate between interior and exterior alpha-cells, we calculate using vector arithmetic the minimum distance between each alpha-cell centroid and its containing contour’s edge. Alpha-cells with a distance of above a predetermined threshold are deemed interior alpha-cells and counted separately (Figure 1). The intensity threshold was determined to be 50, as determined by the above mentioned grid search.

### Pancreatic islet imaging methods

C57BL/6J mice obtained from Jackson Laboratories were fed a normal chow diet or a specific high fat high glucose (HFG) diet where mentioned. Rhesus primates (*Macaca mulatta*) were continuously housed at the NIH Animal Center (Poolesville, MD, USA). The animal center is fully accredited by the American Association for Accreditation of Laboratory Animal Care, and all procedures were approved by the Animal Care and Use Committee of the NIA Intramural Program. Normal and diabetic female primate pancreata were used in this study. For human samples, Dr. Frederic B. Askin from the Department of Pathology at The Johns Hopkins University School of Medicine (Baltimore, MD, USA) provided anonymous human pancreata sections in paraffin blocks.

After sectioning of pancreatic tissue, immunohistological detection of alpha- and beta-cells was achieved with antigen retrieval and incubation with insulin (1:300; Sigma) and glucagon (1:1000; Sigma) antibodies diluted with 1% BSA overnight at 4°C. After washing, sections were incubated for 1 h in fluorescent secondary antibodies (Alexa 488, Alexa 568, 1:1000, Invitrogen). No fluorescent staining was observed in any sections when primary antibodies were omitted. Images were collected using an LSM-710 confocal microscope (Carl Zeiss MicroImaging, Thornwood, NY, USA; Kim et al., 2011).

### Input image processing for *Pancreas*++

Correct formatting of input images for *Pancreas*++ is vital for accurate quantification results. Variations in the size of the input image, for batch or individual analyses, may result in numerical discrepancies in the user output results. *Pancreas*++ takes 256 × 256 .bmp, .jpg, .png, .gif, and .wbmp images. Images not of this size must be scaled using a program such as Adobe Photoshop. Images such as .tifs which are not supported by *Pancreas*++ can be converted to one of the supported file formats using a third party image conversion program such as Pixillion (http://www.pixillion.com/) or an online resource such as convertmyimage.com. It is of crucial importance that alpha-cells are green, beta-cells are red, and all else neither green nor red.

### Program validation methods

Since manual counting of all pixels is excessively labor-intensive and in contrast quantifying total alpha-cells is computationally trivial, technological validation was performed upon the manual quantification of interior alpha-cells. Since interior alpha-cell count is dependent upon both accuracy of cell selection and accuracy of the containing contour, it serves as the best indicator for overall algorithm validity.

Quantification of pancreatic islet size, alpha-cell numbers, alpha-cell size, alpha-cell percentage, and beta-cell percentage was performed with *Pancreas*++, manual counting assisted by NIH-Image J and also with our previously described MATLAB (MathWorks)-based processing toolbox (Kim et al., 2011). For our manual method assisted with Image J, the contour of each islet was drawn and the area was measured. Color images were split into binary positive/negative data using a constant threshold limit, and alpha-cell area was measured. For quantification using our MATLAB (MathWorks)-based process, the region of interest (ROI) was drawn around each islet after background subtraction. The pixels within the bounds of the ROI and above the set threshold of eight were selected, from which actual islet area was calculated. The normalized variance of the ROI was used to calculate an artificial ellipse from which the major and minor axes were determined. Islet morphometry and sizing analyses were performed in an unbiased, random fashion.

## Results

### Description of the user interface

**Figure 2:**
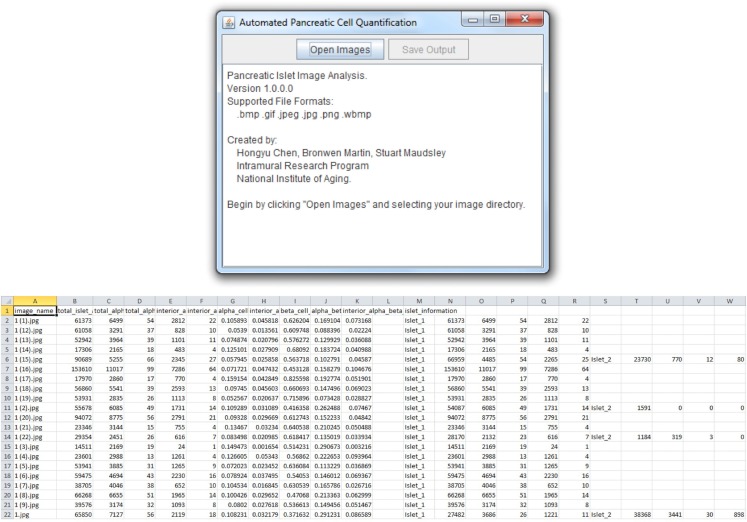
**User interface and output features**. *Pancreas*++ output features include (left to right): image name, total islet area, total alpha-cell area, total alpha-cell count, interior alpha-cell count, interior alpha-cell area, alpha-cell percentage, interior alpha-cell percentage, beta-cell percentage, alpha-cell to beta-cell ratio, interior alpha-cell to beta-cell ratio, and individual islet information. Data can be easily accessed as the output is formatted for analysis in Excel™ or OpenOffice Spreadsheet.

The *Pancreas*++ algorithms previously outlined (see Materials and Methods) and a cross-platform “front-end” interface were implemented using Java. *Pancreas*++ is able to process large amounts of microscopy images in an efficient manner. A user selects the input directory, and all images within the directory are automatically loaded into the program (Figure 2). After processing, the user can save the images into a .csv output file that can be opened using Microsoft Excel™ or any other text editor. The output file contains a table with the image names, total islet area, total alpha-cell area, total alpha-cell count, interior alpha-cell area, interior alpha-cell count, alpha-cell percentage, interior alpha-cell percentage, beta-cell percentage, alpha-cell to beta-cell ratio, interior alpha-cell to beta-cell ratio, and individual islet information with respect to all the aforementioned quantifications. Pancreas++ can be downloaded free of charge at the following address: http://www.irp.nia.nih.gov/bioinformatics/pancreas++.html

### Performance

Our software requires sufficient amounts of free computer memory to compute, e.g., for 512 × 512 images, the program requires about 467 MB of random access memory. The program typically processes about four 512 × 512 images per second using 64-bit Windows 7, a 2.40 GHz processor, and 8GB of random access memory. The time-complexity of the algorithm outlined above is O(*n*^3^).

### Validation of accuracy

**Figure 3:**
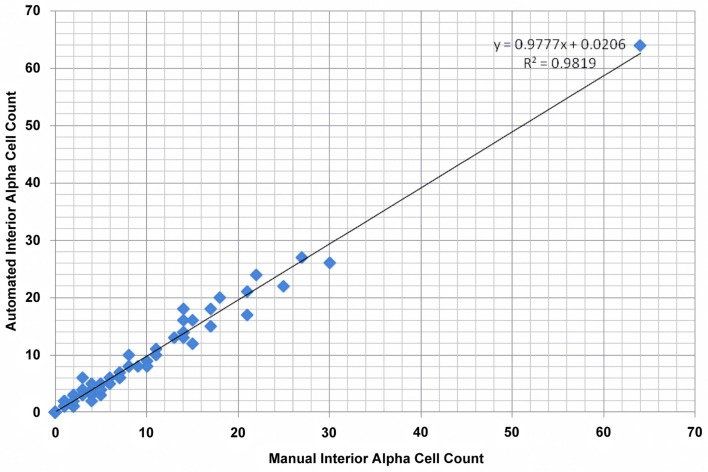
**Validation results for quantification of interior alpha-cells**. Total size of validation set was 75. The Pearson’s correlation coefficient, *p*-value from Pearson’s Chi-square goodness of fit test, average absolute error, and average relative error were 0.9909, 1.7 × 10^−7^, 0.8310, and 0.0158, respectively.

To validate our algorithm’s computational accuracy, *Pancreas*++ was tested on 75 microscopy images. The images were taken to represent a dataset with a high image content variance to test the program’s robustness (“image resilience”). As a comparative reference, manual quantification of the interior islet alpha-cells was also performed. Disambiguation between exterior and interior islets during manual quantification was subjectively determined. The manually counted results were then compared to the results obtained by our algorithm for validation purposes. The results from the comparison are reported in Figure 3. Figure 3 displays a scatterplot of the interior alpha-cell count computed by both automated and manual methods. The slope and intercept coefficients obtained by linear regression are 0.9777 and 0.0206, respectively. The above-zero value of the intercept coefficient indicates a slight overestimation of interior alpha-cells on average. The Pearson Correlation coefficient was computed to be 0.9909, indicating a high degree of correlation between the two methods. A Chi-square test for goodness of fit yielded a *p*-value of 1.7 × 10^−7^, further indicating a deterministic relation between manual counting and the proposed algorithm. The average absolute and relative errors between the two methods were 0.8310 and 0.0158, respectively. Therefore, our automated method can reproduce the accuracy of an experienced molecular biologist but in a mere fraction of the time required. *Pancreas*++ was able to generate nuanced (e.g., calculation of interior alpha-cell counts and interior alpha-cell to beta-cell ratios) and accurate numerical pancreatic islet cellular data in a matter of seconds, compared to manual counting that requires hours of dedicated viewing.

### Application in biological experiments

**Figure 4:**
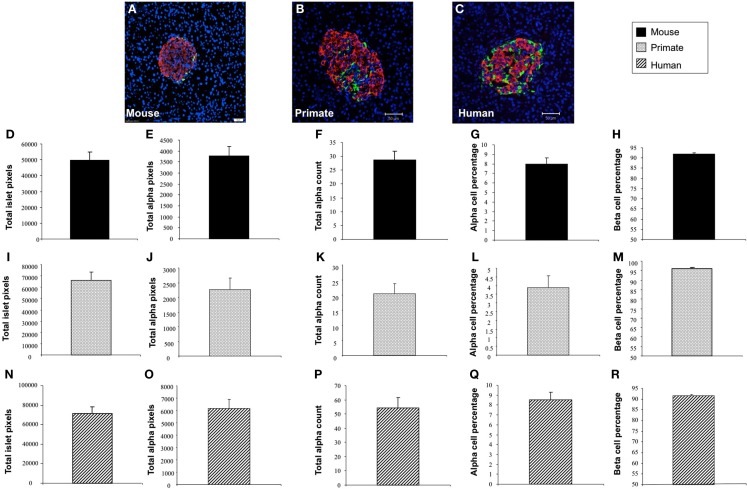
**Application of *Pancreas*++ in mouse, primate, and human pancreatic islet images**. **(A–C)** Are representative images of mouse, primate, and human pancreatic islet images respectively. **(D)** Total islet area of mouse pancreatic islet images (arbitrary units); **(E)** total alpha-cell area of mouse pancreatic islet images (arbitrary units); **(F)** total alpha-cell numbers of mouse pancreatic islet images; **(G)** alpha-cell percentage of mouse pancreatic islet images; **(H)** beta-cell percentage of mouse pancreatic islet images; **(I)** total islet area of primate pancreatic islet images (arbitrary units); **(J)** total alpha-cell area of primate pancreatic islet images (arbitrary units); **(K)** total alpha-cell numbers of primate pancreatic islet images; **(L)** alpha-cell percentage of primate pancreatic islet images; **(M)** beta-cell percentage of mouse pancreatic islet images; **(N)** total islet area of human pancreatic islet images (arbitrary units); **(O)** total alpha-cell area of human pancreatic islet images (arbitrary units); **(P)** total alpha-cell numbers of human pancreatic islet images; **(Q)** alpha-cell percentage of human pancreatic islet images; **(R)** beta-cell percentage of human pancreatic islet images. Data are means ± SEM.

**Figure 5:**
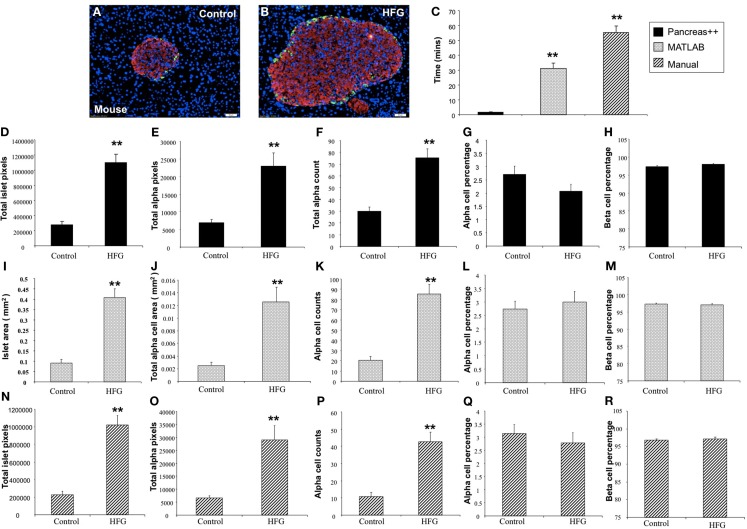
**Analysis of pancreatic islets of mice on control and high fat high glucose (HFG) diet using *Pancreas*++, MATLAB, and manual method**. **(A,B)** Are representative images of pancreatic islet from mice on control and HFG diets respectively. **(C)** Shows the time that *Pancreas*++, MATLAB, and manual method take to analyze the same images. The mice on HFG diet had significantly higher total islet area **(D)**, total alpha-cell area **(E)**, total alpha-cell numbers **(F)**, but similar alpha-cell percentage **(G)** and beta-cell percentage **(H)** compared to the mice on control diet analyzed by *Pancreas*++. Islet area **(I)**; total alpha-cell area **(J)**; total alpha-cell numbers **(K)**; alpha-cell percentage **(L)**; beta-cell percentage **(M)** of mice on control; and HFG diet analyzed by MATLAB showed the same pattern as the results analyzed by *Pancreas*++. Also islet area **(N)**; total alpha-cell area **(O)**; total alpha-cell numbers **(P)**; alpha-cell percentage **(Q)**; beta-cell percentage **(R)** of mice on control and HFG diet analyzed by manual methods showed the same pattern as the results analyzed by *Pancreas*++. Data are means ± SEM. **p ≤ 0.01, *n* = 10/group.

**Figure 6:**
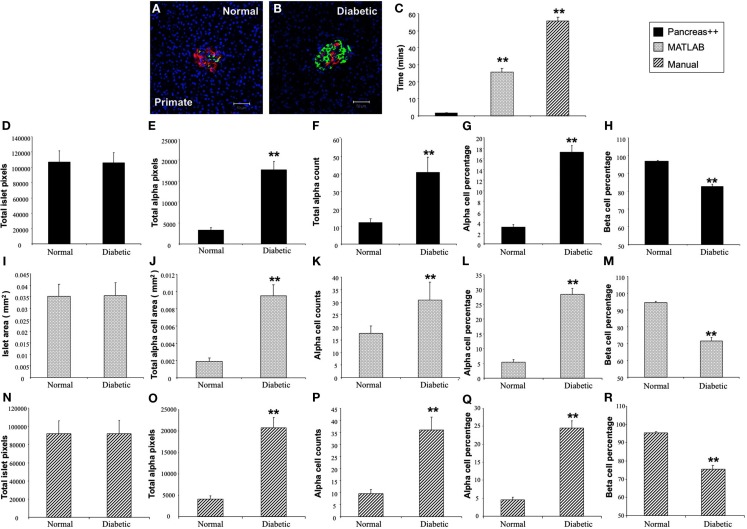
**Analysis of pancreatic islets from normal and diabetic primates using *Pancreas*++, MATLAB and manual method**. **(A,B)** Are representative images of pancreatic islets from normal and diabetic primates respectively. **(C)** Shows the time that *Pancreas*++, MATLAB, and manual method take to analyze the same images. The diabetic primate had significantly higher total alpha-cell area **(E)**; total alpha-cell numbers **(F)**; alpha-cell percentage **(G)**, lower beta-cell percentage **(H)**, but similar islet area **(D)** compared to the normal primate analyzed by *Pancreas*++. Islet area **(I)**; total alpha-cell area **(J)**; total alpha-cell numbers **(K)**; alpha-cell percentage **(L)**; beta-cell percentage **(M)** of normal and diabetic primate analyzed by MATLAB showed the same pattern as the results analyzed by *Pancreas*++. Also islet area **(N)**; total alpha-cell area **(O)**; total alpha-cell numbers **(P)**; alpha-cell percentage **(Q)**; beta-cell percentage **(R)** of normal and diabetic primates analyzed by manual methods showed the same pattern as the results analyzed by *Pancreas*++. Data are means ± SEM. **p ≤ 0.01, *n* = 10/group.

In addition to application with murine islet images, we also tested the performance of *Pancreas*++ with non-murine pancreatic images, e.g., primate and human (Figure 4). In a similar manner to its performance with murine islets, *Pancreas*++ was able to extract islet morphology and cell type specific information from both primate and human images. In biomedical experiments, the comparison of treated group with un-treated control group or the pathological tissue with normal tissue is of vital clinical and experimental importance. In order to demonstrate the accuracy and efficiency of *Pancreas*++ in a pathophysiological setting, we used different methods to quantify and compare pancreatic islet morphology of mice fed a control chow diet with ones fed a deleterious HFG diet (Figure 5). HFG diets cause a metabolic shift from euglycemic states to pathophysiological conditions associated with Type II diabetes. In this pathological state we compared the speed of multiple image analysis between *Pancreas*++, Image J-assisted manual counting, and our MATLAB-based process (20 images in each group were analyzed). We found that for total information processing for the input images *Pancreas*++ was significantly (*p* < 0.01) faster than the other two approaches (Figure 5C). As shown in Figures 5D–H (using *Pancreas*++), the mice fed with HFG diet had significantly increased pancreatic islet size, alpha-cell size, alpha-cells, but similar alpha-cell percentage and beta-cell percentage respectively compared with mice fed with the control diet. The same pattern of islet morphological differences between these two groups was also obtained by quantifying the same images using the MATLAB (Figures 5I–M) or the manual method (Figures 5N–R). The accuracy and efficiency of *Pancreas*++ was also assessed by quantifying and comparing normal and diabetic primate pancreatic islet images (Figure 6). Diabetic primates exhibited significantly increased alpha-cell numbers (Figure 6F), total alpha-cell size (Figure 6E), alpha-cell percentage (Figure 6G), decreased beta-cell percentage (Figure 6H), but similar islet size (Figure 6D) compared with normal primates. The same pattern of differences between normal and diabetic primates was also obtained by quantifying the same images using the MATLAB (Figures 6I–M) or the manual counting method (Figures 6N–R).

## Discussion

In creating *Pancreas*++, we have developed a novel method for the fully automated quantification of islet area, alpha-cell area, quantity, and beta-cell percentage based on pancreatic microscopy images. The proposed algorithm uses active contour models to quantify images accurately and quickly, resulting in an output in an easy-to-read tabular format. *Pancreas*++ can distinguish between relevant pixels and noise, process multiple islets within the same image, and function without the aid of user interaction. The results from the program were validated against the “*gold standard*” of manual counting of interior islets. Results from the validation suggested that while significantly reducing the quantification time compared to manual counting, a high degree of correlation to this standard procedure and a very low error rate was generated. Our novel algorithm allows biologists to not only quantify cell count and area, but also to detect the presence of interior alpha-cells, an indicator of the potential pathophysiological abnormality of a murine pancreatic islet. We also demonstrated the application of *Pancreas*++ in biological pancreatic experiments with divergent species, e.g., primate and human. Our program was able to generate the same results as those obtained using either the MATLAB-based program or with manual counting assisted with Image J. However the batch processing time of *Pancreas*++ to obtain total islet area, total alpha-cell area, total alpha-cell count, interior alpha-cell area, interior alpha-cell count, alpha-cell percentage, interior alpha-cell percentage, beta-cell percentage, alpha-cell to beta-cell ratio, and interior alpha-cell to beta-cell ratio was significantly less than the other approaches tested. In these tests even with the increased processing speed, no significant loss of information retrieval accuracy was noted. Automated quantification algorithms greatly reduce user bias and allow biologists to rapidly process large amounts of biomedical images. However it is prudent for researchers already employing a different islet quantification process to personally validate, using the “*gold standard*” of manual counting, their own data with the automated output of *Pancreas*++. We therefore recommend a thorough “in-house” validation and quality control of image size, pixel density, dye selection, confocal microscope settings and image type before any large-scale, automated implementation of *Pancreas*++ in a new experimental setting. Our novel algorithm allows biologists to not only quantify cell count and area, but also to detect the presence of interior alpha-cells, an indicator of the potential abnormality of a pancreatic islet in murine tissue. Accurate, unbiased extraction of information on both the quality and quantity of endocrine cells in microscopy images may help scientists develop an increased understanding of metabolism and metabolic disorders in the future.

## Conflict of Interest Statement

The authors declare that the research was conducted in the absence of any commercial or financial relationships that could be construed as a potential conflict of interest.
